# 

**Published:** 1916-04-22

**Authors:** 


					Interesting Bequest to Edinburgh University.
The late Sir Alexander R. Simpson, for many years
professor of midwifery in the University of Edinburgh,
has left to the University the museum formed by his
uncle, the late Sir James Simpson, the discoverer of
chloroform as an anesthetic.
St, George's Hospital.
Sir Gerard Lowther, one of the treasurers, "who
retired, we regre?t to learn, on account of ill-health, and
whose death has since been reported, is succeeded by
Lord Newton. The number of the visiting staff, which
is in peace time twenty-five, has been reduced to six-
teen; of the remainder ten are holding commissions or
doing work in other war hospitals; 445 sick and wounded
sailors and soldiers have been treated in the wards. It
has been arranged for the hospital to take over the
management of the St. George's Hospital Institute of
Trained Nurses, but the accounts will be kept separate
and no part of the profits used for hospital purposes. The
ordinary expenditure of the hospital exceeded the ordi-
nary income by ?11,296, but the legacies received
amounted to ?14,868. It is stated that the year's de-
ficiency " was met partly by utilising legacies received
during the year, by the sale of ?20,045 Midland Railway
2^ per cent, debenture stock, which realised ?12,199, and
by an overdraft allowed by the bankers." As the sale
of stock was in itself more than sufficient to make up
the deficiency there must have been some further reason
for the financial operation described in the paragraph
quoted. The cost per occupied bed has risen from ?110
to ?124.
It is our invariable practice to pay for all
items of news which we may publish. The minimum
payment is 5s. Paragraphs of about twenty lines
in length?that is to say, of 150 words or less?
are preferred. In this section during the_ war we
propose to give 10s. to the correspondent who may
send us in any week the best incident connected
with hospital life and work. In this way we hope
to afford to all practical aid and encouragement;
while those who reciprocate will be able to render
us invaluable service. Contributions should be
addressed to The Editor, The Hospital, 28 and 29
Southampton Street, Strand, London, W.C., and,
to be in time for the current week's issue, should
reach him by the first post on Mondays.
For Matrons' and Sisters' Appointments see page 92.
Matrons' and Sisters'
Appointments.
Benfleet Hall Auxiliaky Military Hospital, Sutton.?
Miss I. G. Kent has been appointed night superintendent.
Trained at St. Mary's Hospital, she was later a member
of the private staff of the Royal County Hospital, Win-
chester, and has acted as sister at Lady Lytton's Hospital,
Nottingham Place.
Moor Edge Hospital for Sick Children, Newcastle-on-
Tyne.?Miss Lilian Doxey has been appointed sister. She
was trained at the Children's Hospital, Sheffield, after-
wards becoming charge nurse there and later sister at
Wallasey Fever Hospital, Liscard.
NOTE.?Particulars of Appointments for insertion in this
column wlli be welcomed.
HOSPITAL RESULTS IN 1915.
More Early Reports of the Voluntary Hospitals.
East London Hospital for Children.?Many annual
reports supply photographs of modern hospital waTds,
but this is the first we have seen this year containing a
picture of what a ward should not be. The illustration
in question is of a ward (or perhaps the ward) of the
original hospital in 1872. Including legacies of ?1,200
the total income for 1915 was ?11,783, and the expendi-
ture was ?11,125; this turn in the tide of financial ill-
luck enables the board of management to reduce the debt
of about ?5,000. We notice that gifts in kind from
Sydney," valued at ?229, were received during 1915.
There is a marked decrease in annual subscriptions.
Torbay Hospital, Torquay.?The information sup-
plied by the seventy-second annual report starts straight
away on the front cover, where the names of the chief
office-bearers of the institution are detailed; moreover,
the illustrations are printed on each side of plate-paper,
these facts showing that the executive is imbued with
the economical spirit of the times. From the photo-
graph of the "Louisa Cary" ward we gather that gas
for lighting purposes is still used. The in-patients have
increased from 668 in 1913 to 1,056 in 1915; 425 sailors
and soldiers were treated in the wards during the year
under review. The total income was ?6,974 (?2,024 from
patients' payments) and the expenditure ?6,901.
Royal London Ophthalmic Hospital.?The effects
of the move from Moorfields to the City Road seventeen
years ago find an echo in each annual report, and the
difference between a freehold building, lightly rated by
the municipal authorities, and the burden of a large
ground-rent and a heavy assessment for rates is a financial
consideration adding substantially to the responsibilities
of the committee of management. The balance to the
bad of income and expenditure last year was not a large
one?only ?402?but as there has been an annual deficiency
for four successive years the aggregate result is ?2,196.
Twenty beds are devoted to the needs of wounded
soldiers, and 2,137 were last year treated in the out-
patient department.
Pontypool and District Hospital.?The working men
of Pontypool and the surrounding district should be
proud of their hospital, for they provide a large part of
the sum needed for its maintenance and share in the
actual management, being well represented on the execu-
tive board and in the list of governors. The total
ordinary income of the institution for 1915 was ?3,121,
and of this sum ?2,002 was received in workmen's contri-
butions ; as the total ordinary expenditure was only
?2,365 the, executive has little anxietv on the score of
ordinary expenditure. During the past year a new
nurses' home and a children's ward have been opened.
The annual report is presented in an attractive form, with
plenty of interesting illustrations.
Norfolk and Norwich Hospital.?The Lord Mayor
of Norwich presided at the annual meeting on April 8.
It was reported that 3,200 wounded soldiers had been
received since the commencement of the war, and that
during 1915 ?12,185 had been contributed by the War
Office towards the maintenance of wounded soldiers,
amongst whom the death-rate had been 0.8 per cent.
The total receipts for the year were ?29,853, a sum two
and a half times greater than the income of the previous
twelve months. The total income exceeded the expendi-
ture by ?3,416, and an overdraft at the bank was reduced
by that sum. An appeal had been issued,, taking the
form of 200 written letters and costing one sovereign, for
contributions of ?5 per month for each of the soldiers'
beds, and the response brought in ?5,721 to the hospital
exchequer. The beds were maintained by individuals
and groups ,of persons of all classes and denominations;
domestic servants contributed no less than ?152. In
spite of the largely increased expenditure, two gratifying
facts remained : firstly, that a new source of income had
been reached, and, secondly, that the generosity of the
public had exceeded the most sanguine expectations. The
annual report states that Mr. Frank Inch, the new secre-
tary, has now taken up his duties, and a pleasing refer-
ence is made to the work of the late secretary, Mr.
Frank Hazell, now superintendent and secretary at the
Manchester Royal Infirmary. Norfolk is in what may
be described as " a Zeppelin district," and the following
paragraph from the report illustrates the strange condi-
tions under which the inhabitants of the Eastern Counties
live at the present time : To conform with the Lighting
Orders, which become more and more stringent, has
proved a very great difficulty, as it has been impossible
to provide adequate roller blinds for the numerous
windows (upwards of twelve hundred). When all lights
have been extinguished during an enemy air raid, the
work has been carried on by means of hurricane lanterns,
and the difficulty of receiving a convoy under such con-
ditions can be well imagined.
Batbs Of Subscription : Twelve months, 6/6; Six months, 4/-; Th ree months, 2/-, post free to any address in the United Kingdom.
Abroad, Twelve months, 8/8; Six months, 5/-; Three months, 2/6, post free.?Address The Subscription Dept., " The Hospital,"
Tho Hospital Building, 28/29 Southampton Street, Strand, London. W.O.
NATIONAL RELIEF FUND.
Treasurer?H.R.H. The Prince of Wales.
Will every reader of "THE HOSPITAL" send at
least Is.?
To H.R.H. The Prince of Wales,
Buckingham Palace, London.
I beg to enclose ? : s. d. as a donation to
the National Belief Fund.
Name ...
Address
The envelope containing this coupon need not be stamped.

				

